# Environmental impact of a radiology department: greenhouse gas emissions and primary energy accounting and strategies for sustainable practice

**DOI:** 10.1186/s13244-026-02436-9

**Published:** 2026-10-15

**Authors:** Stefania Rizzo, Christoph Rüesch, Marco Budin, Rolf Wyttenbach, Ermidio Rezzonico, Filippo Del Grande

**Affiliations:** 1 Department of Radiology, Institute of Integrated Diagnostic of Southern Switzerland (IDISI), Ente Ospedaliero Cantonale (EOC), Lugano, Switzerland; 2 Faculty of Biomedical Sciences, Università della Svizzera italiana (USI), Lugano, Switzerland; 3 Enertel GmbH, Grono, Switzerland; 4 University of Bern, Bern, Switzerland

**Keywords:** CT, GHG emissions, MRI, Primary energy, US

## Abstract

**Objectives:**

To assess the environmental impact of a radiology department’s operations (primary energy consumption and greenhouse gas emissions (GHG); to identify the main levers for reduction.

**Materials and methods:**

Data collection included measured and calculated energy consumption of imaging machines (2 MRI, 2 CT, 1 interventional radiology (IR) suite, 2 US, 3 radiography machines), workstations, lighting, ventilation; cooling and heating for imaging systems and rooms; materials/pharmaceuticals; external services (e.g. laundry and information technology (IT)). The electrical energy consumption of building systems, lighting, and IT equipment was primarily determined through measurements; other energy consumption figures were estimated using technical data and meteorological data. For the calculation of GHG emissions and primary energy consumption, a specialised tool using specific conversion factors from established environmental databases was developed.

**Results:**

Our hospital switched from coal-based electricity to renewable electrical energy (hydropower), leading to a reduction in primary energy consumption (−64%) and GHG emissions (−92%) of the radiology department. Following this change, without a reduction in direct energy consumption, the MRI systems showed a marked reduction in primary energy consumption, although still having the highest primary energy consumption per exam (49.2 kWh compared to 13.9 kWh (CT1) and 48.9 kWh (IR)). After the transition to renewable energy, the major source of GHG emissions shifted from electrical energy toward materials/pharmaceuticals.

**Conclusion:**

Our findings support the transition to renewable energy sources for electricity, as a key strategy for reducing GHG emissions and primary energy demand of radiology departments. Following this shift, selecting materials and pharmaceuticals with lower emissions and primary energy factors can further mitigate the environmental impact.

**Key Points:**

***Question*** Radiology departments are significant sources of energy consumption and GHG emissions.

***Findings*** Adoption of renewable energy reduces primary energy consumption and GHG emissions.

***Critical relevance statement*** GHG emissions in a radiology department can be significantly reduced by transitioning to renewable energy sources. After this shift, emissions will mainly depend on the materials and pharmaceuticals used, such as disposable supplies and contrast agents.

**Graphical Abstract:**

**Figure:**
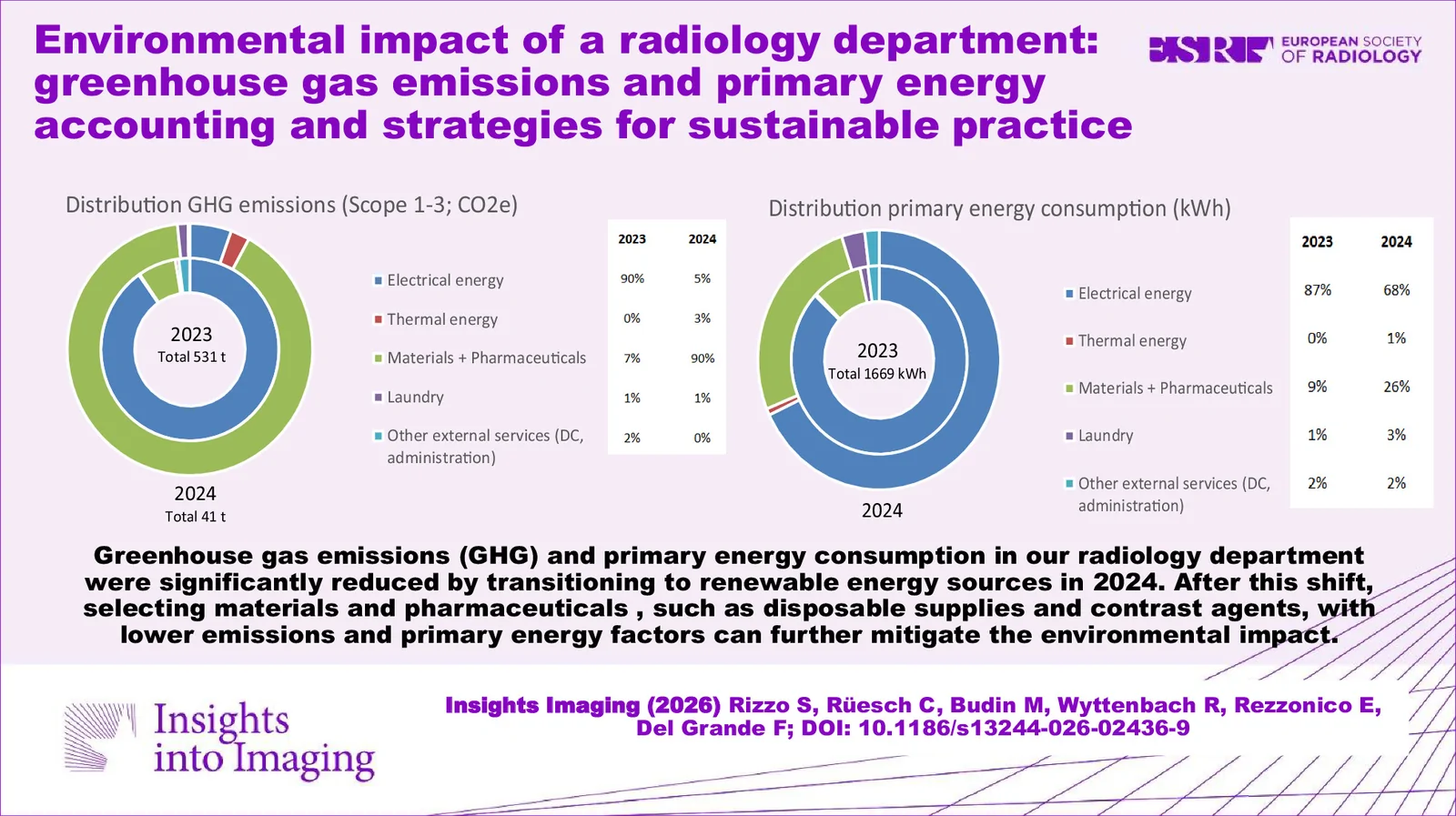

## Introduction

Healthcare systems are major contributors to environmental impact, accounting for approximately 4–10% of global greenhouse gas (GHG) emissions [1–3]. Within hospitals, radiology departments are major sources of energy consumption, material use, and waste generation, and therefore also of GHG emissions. Indeed, radiology departments house energy-intensive equipment, such as MRI, CT, interventional radiology (IR) suites, ultrasound systems and digital radiography devices [4, 5], which require substantial electrical power, complex cooling and support infrastructure, further amplifying their environmental footprint.

Primary energy consumption refers to the sum of direct energy (energy consumption that can be directly measured) and indirect energy (energy required throughout the upstream and downstream stages of the value chain). On the other hand, the GHG emissions and the primary energy consumption of a radiology department include not only major imaging equipment, but also building infrastructure, information technology, consumables and pharmaceuticals [6]. A recent scoping review summarised the environmental impact of radiology departments, highlighting that MRI and CT are the most energy-intensive modalities. Reported annual CO₂-equivalent (CO₂e) emissions per scanner range from 53.1 ± 13.2 metric tons for a single MRI to 12.6 ± 2.9 metric tons for CT, and per-scan emissions range between 6.2–76.2 kg for MRI and 1.1–13.4 kg for CT, depending on system limits, methodology and utilisation [3]. Esmaeili et al estimated the carbon footprint of a single MRI examination to be 22.4 kg CO₂e per patient. In-hospital energy consumption, including the MRI machine, ancillary devices, and lighting, amounted to 29 kWh per patient, whereas out-of-hospital energy use, encompassing fuel for electricity generation and transmission, as well as the energy required to produce disposable, consumable, and reusable products, was measured at 75 kWh per patient [7]. A recent life-cycle assessment (LCA) of a radiology department reported 4.6 kt CO₂e emissions over ten years, with MRI and CT machines responsible for 48% and 24% of cumulative emissions, respectively [4]. Furthermore, since CT and MRI energy consumption is substantial, previous studies have already demonstrated that there is potential for significant energy and cost savings during nonproductive idle and system-off modes in modern scanners [8].

However, the existing literature has not fully captured the comprehensive environmental impact of a radiology department as an organisational unit embedded within a broader hospital system, including the assessment of primary energy consumption and GHG emissions that are crucial for identifying effective mitigation strategies [9].

Therefore, the purpose of this study was to conduct a thorough assessment of the primary energy consumption and GHG emissions, as key metrics for assessing the environmental impact of a radiology department, with the ultimate goal of identifying key areas for mitigation and moving towards a sustainable radiology practice.

## Materials and methods

This is an observational retrospective monocentric study, not involving human participants, therefore, no IRB approval was required.

### Systems and boundaries

Throughout the entire study, the same system boundaries, methodology, and consumption categories were applied. Consequently, all data and results presented are based on a consistent methodological framework.

#### Inclusion criteria

This study included all imaging systems of the radiology department of one regional hospital, including one MRI 3.0 T (Philips, Ingenia 3.0 T), one MRI 1.5 T (Philips Ambition X 1.5 T), two CT (Siemens Somatom X.Ceed), one IR suite (Philips Allura Xper FD 20), two US (GE Loqiq) and three conventional radiography machines, including fluoroscopy (2 Philips Digital Diagnost C90 HP; 1 Celex, NRT x Ray, Leuag AG). One of the two CT systems (CT 1) and the 2 MRI systems are shared with the neurology department, whereas the other CT system (CT 2) is shared with the emergency department. The allocation between our department and the other departments was based on the number of examinations performed, and these allocation factors were incorporated proportionally into all corresponding calculations and analyses.

#### Exclusion criteria

Mammography machines were excluded, as they are located in a different area of the Hospital. Staff and patient transportation were also excluded, although they have a significant impact, because this issue is being addressed through a hospital-wide strategy.

#### Data collection and analysis

**Fig. 1:**
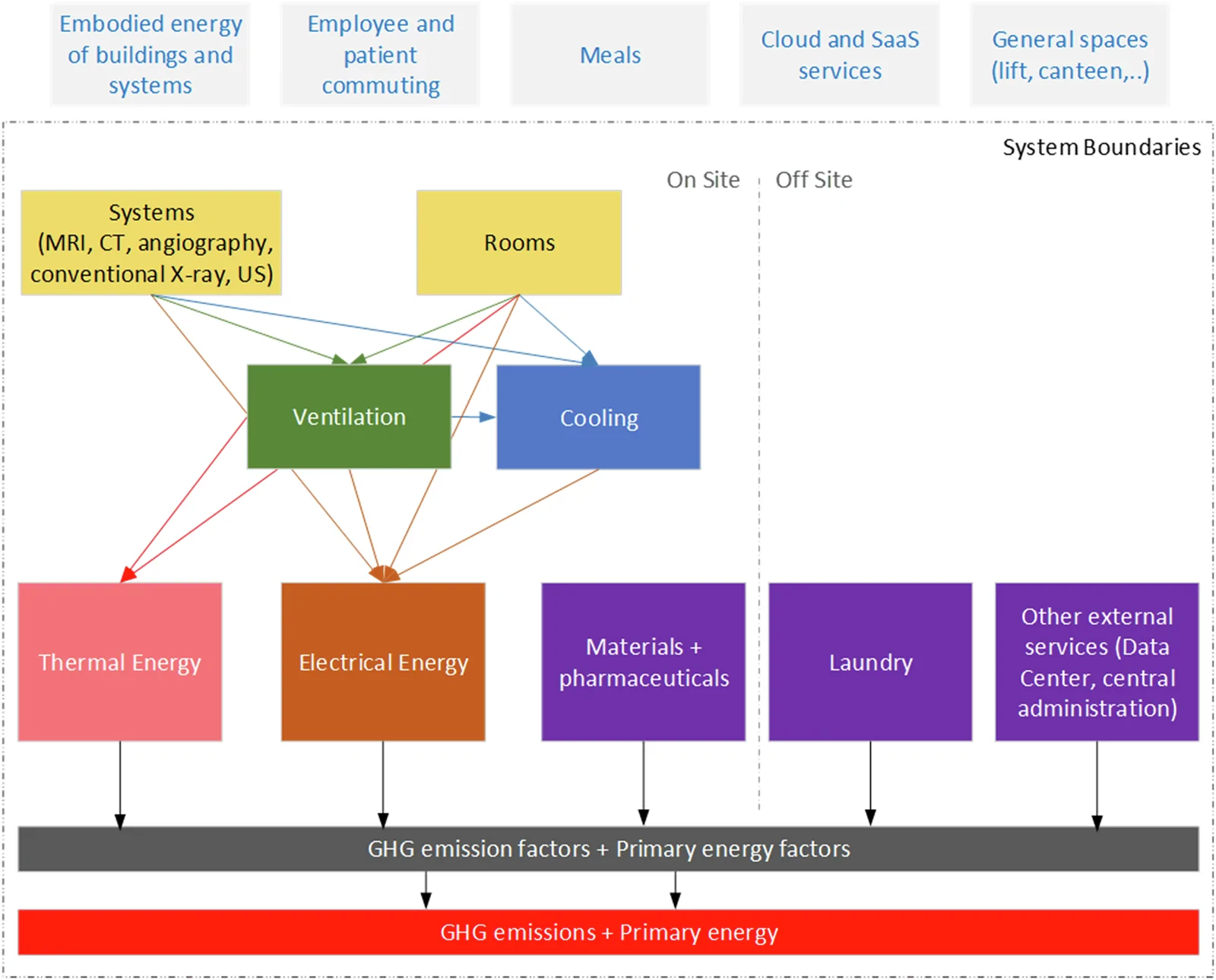
The figure shows the consumption included within the system boundaries and the principle for calculating GHG emissions and primary energy consumption

We encompassed both measured and calculated power consumption for all the above-mentioned imaging systems (Fig. 1). This included energy in the rooms, such as lighting, workstations (including image computing), local network infrastructure, and other general devices. Cooling data for the imaging systems and rooms provided by the hospital’s central refrigeration plant, were included, as well as heating data from the regional district heating network based on waste-to-energy incineration. Fresh air ventilation supplied to the rooms via ducts from a central air-handling unit was also included, as detailed below. Additionally, material and pharmaceutical use, along with external services, such as laundry and information technology (wide area network (WAN)—transmission and storage/retrieval in our two own data centres), were integrated into the assessment. The embodied energy and associated GHG emissions related to the infrastructure and the imaging systems mentioned above, including their construction and dismantling, were not taken into consideration.

### Measurements

#### Primary energy consumption

Most of the direct power consumption of all consumers was determined through power measurements (November 2023–April 2025; approximately 300,000 measured data points), which were continuously recorded using the hospital’s energy monitoring system (Schneider EcoStruxure Power Monitoring Expert). In the absence of suitable measurements, the electricity consumption of the MRI 1.5-T system was determined using a model developed by our group, based on MRI utilisation data and technical specifications provided by the manufacturer. When compared with the measured data, the model deviated by only 3.8% in 2023 and 2.7% in 2024 for the MRI 3.0 T system.

The electrical energy consumption for cooling was estimated based on the electricity consumption of the systems and a typical annual efficiency of the installed cooling production compared to electricity consumption (seasonal energy efficiency ratio (SEER) = 3.33). Ventilation power consumption was calculated based on room-specific air volumes (61% derived from installation plans and 39% estimated from air-change rates according to room type and floor area), including all rooms of the radiology department (technical rooms, administration, meeting rooms, restrooms, and offices), as well as a typical volume flow/power consumption coefficient for the existing installations in accordance with Swiss Society of Engineers and Architects (SIA) standard 382/1 2007 [10].

Heat consumption for secondary rooms, such as offices, was determined based on room layouts, construction characteristics, indoor temperature settings, and monthly average outdoor temperatures.

Primary energy was calculated according to the Swiss definition, using life cycle assessment (LCA)-based factors [11].

The energy consumption associated with PACS that was not already accounted for in the hospital’s energy use was estimated based on our network architecture. The resulting WAN transmission energy values were cross-checked against those reported by Aslan et al [12]. Data storage energy consumption was estimated using the typical power demand of enterprise hard drives in our data centres, including allocations for cooling and other supporting infrastructure. Assuming that each examination is retained in the data centres for 25 years and transmitted three times across the WAN during its lifecycle, the cumulative energy requirement was estimated at 588 Wh per GB of stored data. This value represents the total energy consumption per GB over the entire 25-year retention period and was fully allocated to the year in which the data were initially stored.

#### GHG emissions

GHG emissions were calculated from the above-mentioned data together with emission factors (Scope 1-3) according to Ecoinvent (V.3.7.1, Oct. 2020) for the electricity mix used [13] and values published by the Swiss Federal Office of Energy for 2024 for similar district heating suppliers [14]. The chosen method is consistent with the GHG Protocol Corporate Standard and aligned with EN ISO 14064-1 (a globally recognised standard provided by the International Organization for Standardization (ISO), organised into three parts to cover a robust framework for quantifying, reporting, and verifying GHG emissions) [15].

### Calculations with artificial intelligence (AI) tools

The estimation of GHG and primary energy coefficients for materials and pharmaceuticals represented the greatest challenge. Detailed purchasing records from 2023 were used to determine the quantities of materials and pharmaceuticals for each individual product and to attribute them to each system type (e.g. MRI systems). These consumption figures were then normalised per examination and extrapolated to the year 2024. Indeed, the radiology department used almost 800 different materials and pharmaceuticals, but there were little or no published data available for most of them; LCAs were often available only for packaging materials. Therefore, the sustainability coefficients were estimated using AI assistants (cradle-to-grave, including feedstock energy, packaging and transport to the Hospital) with the following approach. Step 1: clustering and cluster-level approximate calculations. Step 2: detailed calculations for materials and pharmaceuticals with a higher impact (OpenAI ChatGPT 5, Thinking as reasoning mode, 28–30 September 2025). In order to eliminate or greatly reduce the risk of hallucinations, an AI assistant based on a different AI engine (Anthropic’s Claude, 30 September 2025, ClaudeSonnet 4.5) was used to perform control calculations for the elements with a major influence on the results. Lastly, the materials and pharmaceuticals accounting for > 90% of GHG emissions and primary energy consumption in these initial analyses (more than 70 products) were recalculated with a third AI assistant (Google Gemini 3 Pro, Deep Research, 31 December 2025–11 January 2026) (Supplementary Fig. 1). For each product, a report was generated containing the LCA calculations in accordance with ISO 14040, a document provided by the ISO describing the principles and framework for LCA [16] (Supplementary Fig. 2). These calculations were based on specifically referenced sources and included detailed information on the underlying assumptions (e.g. transport routes, vehicles used, energy sources) that were specified in our requests, as well as intermediate and final results, while excluding the training datasets of the AI assistants, to reduce the risk of hallucinations (Supplementary Table 1).

### Tool developed for calculations

A specialised tool was developed for the calculation of GHG emissions and primary energy consumption, which incorporated electricity consumption data from the systems (November 2023–April 2025), data from secondary systems (IT, laundry,) and data on materials and pharmaceuticals acquired in 2024 (Supplementary Fig. 3). Electrical energy data from incomplete years were extrapolated to represent a full 12-month period, as the major systems in our setting (with cooling mainly provided by chillers with constant recooler and cooling setpoints, without free cooling, and with a limited cooling contribution from ventilation) exhibit only limited monthly variation in energy consumption. For plausibility checking, a simplified model was developed, partially based on alternative sources and simplified calculations.

## Results

### Primary energy consumption

Until the end of 2023, the entire hospital, including the radiology department, was supplied with an electricity mix from coal-fired power plants, whereas in 2024, it switched from coal-based electricity to renewable electrical energy from hydropower plants. As the primary energy and GHG emission conversion factors are broadly comparable across the main renewable electricity sources, particularly in comparison with the substantially higher conversion factors of coal-based electricity (coal-based electricity: 1.300 kg CO₂e/kWh and 3.940 kWh/kWh; hydropower: 0.007 kg CO₂e/kWh and 1.190 kWh/kWh; wind power: 0.017 kg CO₂e/kWh and 1.290 kWh/kWh; solar power: 0.098 kg CO₂e/kWh and 1.360 kWh/kWh), throughout this study we refer to renewable electricity, even though all data and results are based on a hydropower electricity mix.

**Fig. 2:**
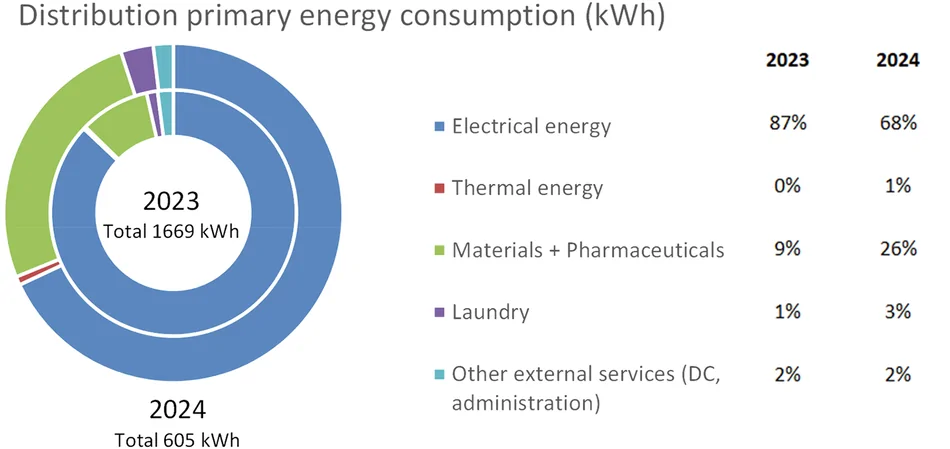
Primary energy consumption in the radiological department in the years 2023 and 2024. The diagram shows that switching from coal-based electricity (year 2023, internal circle) to renewable electrical energy (year 2024, external circle) led to an evident reduction in total primary energy consumption (from 1669 kWh to 605 kWh)

Since the primary energy factors for electricity from coal-fired power plants and renewable sources differ substantially (coal: 3.94 kWh/kWh; hydropower: 1.19 kWh/kWh), this transition led to evident changes in primary energy consumption, as shown in Fig. 2. Accordingly, all systems (including the scanners and all secondary consumers such as cooling) showed a substantial reduction in primary energy consumption (Supplementary Fig. 4) from 2023 to 2024 and 2025.

**Fig. 3:**
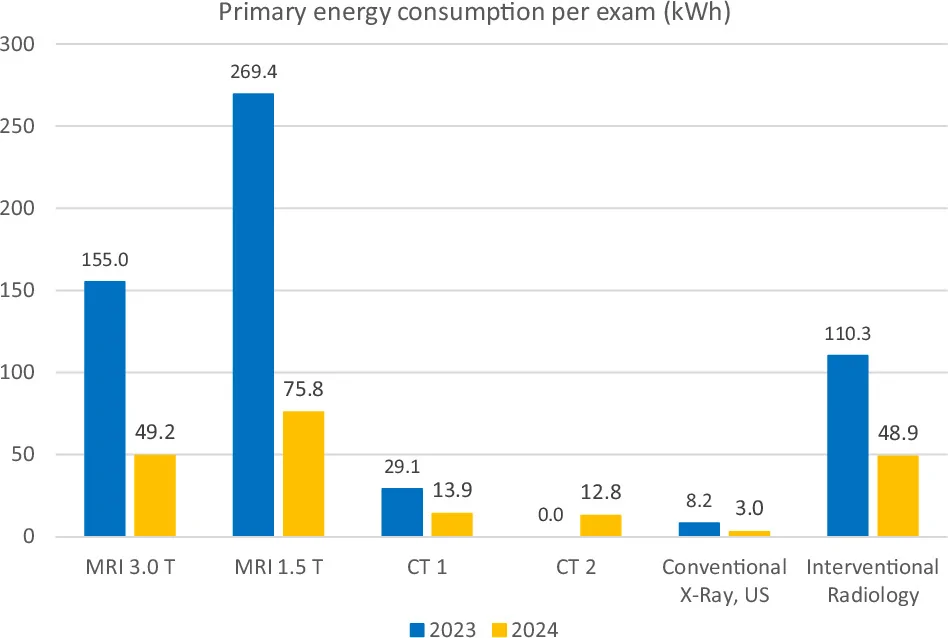
The bar chart shows the primary energy consumption per exam (kWh) of the systems of our radiological department (including all secondary consumptions) during the years 2023 and 2024: although the MRI systems showed a marked reduction in primary energy consumption, they still have the highest energy consumption per exam, compared to CT systems, conventional X-ray, US and IR suite

As shown in Fig. 3, following the transition from coal-based electricity (2023) to renewable energy (2024)—without any reduction of the consumption of direct energy of the systems—MRI systems showed a marked reduction in primary energy consumption (e.g. the 3.0 T machine consumption per exam dropped from 155 kWh to 49.2 kWh), although they still exhibited the highest energy consumption per examination (49.2 kWh per examination, compared with 13.9 kWh for CT1 and 48.9 kWh for the IR suite). Of note, due to operational reasons, the MRI 1.5 T performed significantly fewer examinations than the MRI 3.0 T, leading to higher non-productive consumption (e.g. overnight) per examination and an overall higher primary energy consumption per exam (e.g. in 2024: MRI 3.0 T, 49.2 kWh/examination; MRI 1.5 T, 75.8 kWh/examination).

The median of the daily mean power demand of the MRI 3.0 T (direct electrical consumption) during weekday operational hours (theoretical operating schedule) was 22.00 kW for the period November 2023 to December 2024, whereas the corresponding median outside these hours was 7.50 kW (Supplementary Fig. 5). Direct electrical energy consumption remained relatively stable across the months (Supplementary Fig. 6).

### GHG emissions

**Fig. 4:**
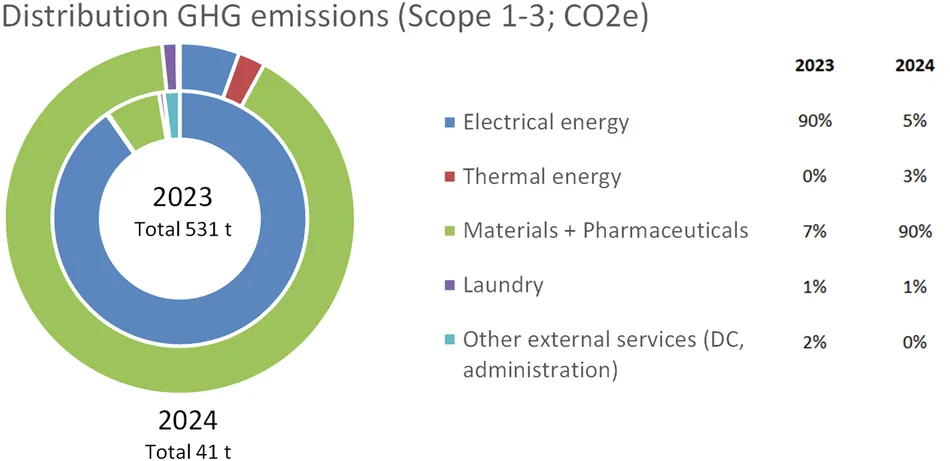
GHG Emissions (scope 1–3) of the radiological department in the years 2023 (internal circle) and 2024 (external circle). The diagram shows that, following the switch to renewable energies (year 2024), the electrical energy consumption (blue part) had no major influence on GHG emissions, compared to the year 2023, whereas the main source of GHG emissions in 2024 shifted clearly toward materials and pharmaceuticals (green part)

As shown in Fig. 4, the transition from coal-based electricity to renewable energy (without any reduction in the consumption of direct energy of the systems) resulted in a marked reduction in the overall GHG emissions of the radiology department (year 2023: 531 t; year 2024: 41 t). While electrical energy had previously been the dominant source of GHG emissions, the major source of GHG emissions in 2024 shifted clearly toward materials + pharmaceuticals. This is attributable to the substantially lower emission factors of renewable energy sources (coal: 1.300 kg CO₂e/kWh; hydropower: 0.007 kg CO₂e/kWh). Other potential sources, such as heat consumption, laundry, and external services (mainly dependent on electrical energy), still show a minor impact.

**Fig. 5:**
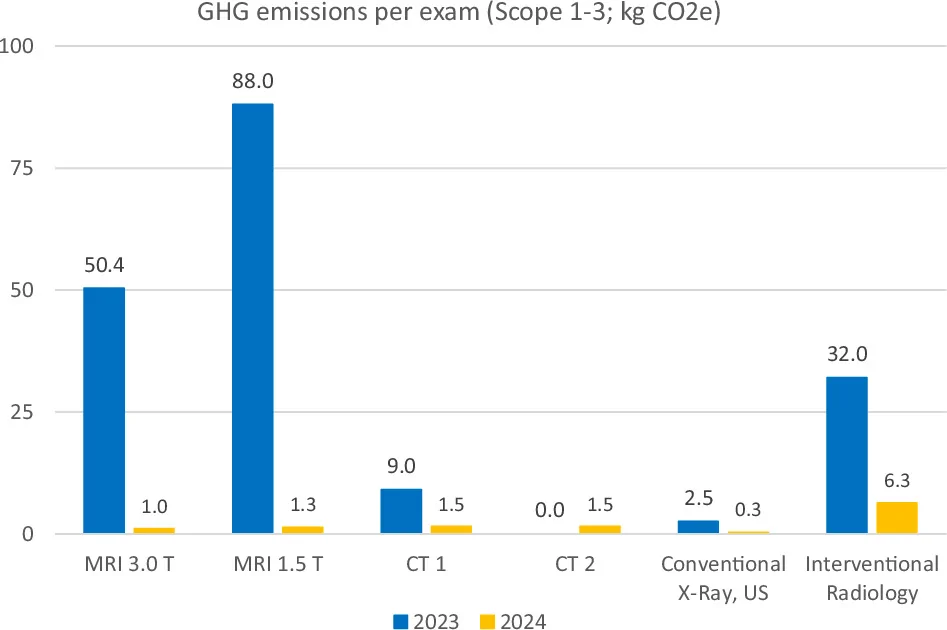
GHG emissions per examination (including energy consumption, laundry, DC, administration, materials, and pharmaceuticals) on the different imaging systems. Following the transition from coal-based electricity (year 2023) to renewable energy (year 2024), GHG emissions per MRI exam dropped significantly, while emissions from CT machines showed a smaller reduction. The emissions of the IR suite per exam remained at a relatively high level and accounted for a relatively major contribution in the year 2024

A simulation showed that, even with a fossil-based non-renewable heating energy source (heating oil), materials and pharmaceuticals had by far the greatest impact (2024: 37 t CO2e for materials and pharmaceuticals vs 17 t CO2e for heating). Under carbon-based non-renewable electricity conditions, MRI systems produced significantly higher GHG emissions per examination than CT systems, whereas after the transition to renewable electricity, their emissions became comparable (Fig. 5). Indeed, the CO_2_e emissions of the MRI systems (including all secondary consumptions) were 50.4 kg/examination (MRI 3.0 T) and 88.0 kg/examination (MRI 1.5 T) in 2023, and 1.0 kg/examination (MRI 3.0 T) and 1.3 kg/exam (MRI 1.5 T) in 2024, respectively. The CO_2_e emissions of the CT 1 system (including all secondary consumptions) were 9.0 kg/examination in 2023, and 1.5 kg/examination in 2024. Notably, the GHG emissions per examination of the IR suite became lower in the year 2024 (6.3 kg/exam) compared to the year 2023 (32.0 kg/exam), but still represented the highest GHG emissions per examination within the department. This result is mainly attributable to pharmaceuticals and materials with a higher environmental impact used during the IR procedures.

Measurements from the first three months of 2025 (not shown in Fig. 5) confirmed the 2024 results.

**Fig. 6:**
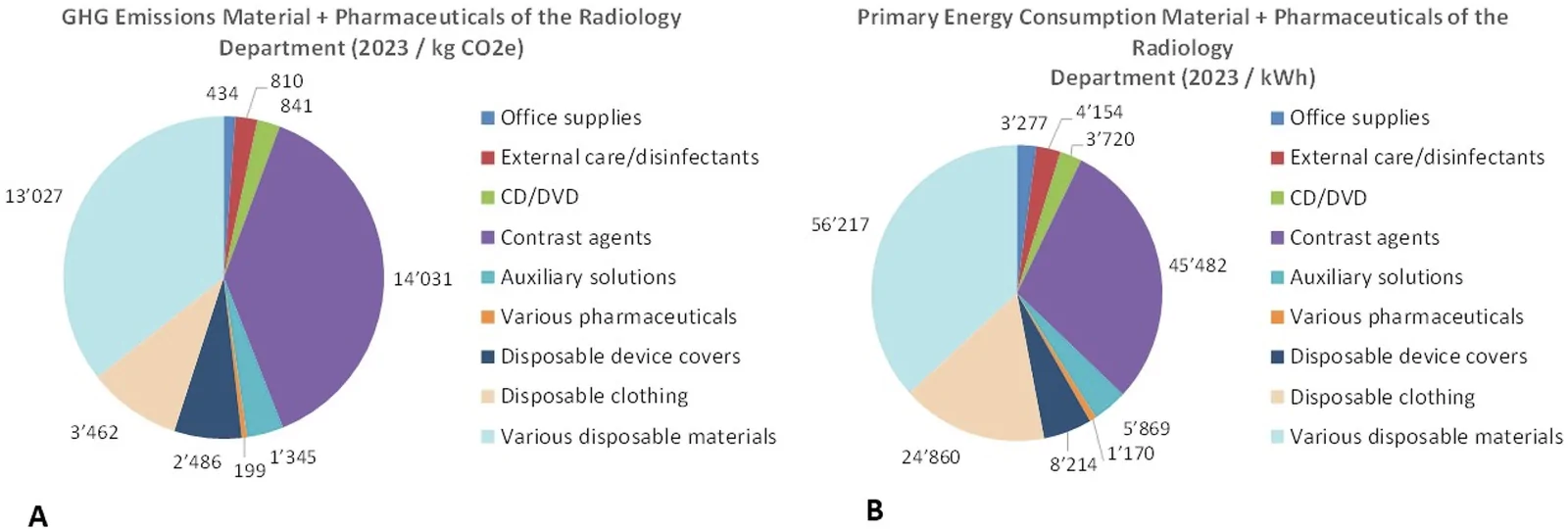
Pie chart of the GHG Emissions (**A**) and primary energy consumption (**B**) from the materials and pharmaceuticals category of the radiology department in the year 2023, shows the relative contributions of each subgroup of materials and pharmaceuticals, as well as the absolute emission and energy consumption values. As shown, contrast agents (purple slice) and various disposable materials (light blue slice) account for the largest share of GHG emissions and of primary energy consumption

A more detailed analysis of material and pharmaceutical consumption confirmed that contrast agents and disposable materials account for the largest share of GHG emissions and primary energy consumption (Fig. 6). Supplementary analyses showed that the emissions and energy demand associated with materials and pharmaceuticals are strongly influenced by the energy mix, transportation, product composition, and system boundaries.

The substantial contribution of materials and pharmaceuticals explains why CT scans in 2024 have slightly higher total GHG emissions per examination than MRI scans. When renewable electricity is used, electricity consumption accounts for only a small proportion of the overall GHG emissions, making the impact of contrast agents a dominant contributor. Consequently, shifting from a fossil-based to a renewable electricity mix results in a significantly greater relative reduction in GHG emissions for MRI than for CT scans.

## Discussion

This study demonstrates that, although the electrical energy consumption from MRI and CT systems is usually considered the largest contributor to GHG emissions in a radiology department, transitioning from coal-based non-renewable to renewable energy sources (hydropower) led to a significant reduction in the carbon footprint of these imaging modalities. The resulting reduction is considerably greater than that achieved through some energy-saving measures such as switching off systems outside operating hours, a practice that had already been implemented in our department several years ago.

Following this shift, the main sources of emissions became materials and pharmaceuticals, particularly contrast agents and disposable materials, underscoring the critical role of sustainable procurement and waste management practices in healthcare environments.

As energy markets in virtually all countries across Europe and the Americas, as well as in the largest economies of Asia, provide mechanisms that separate the physical delivery of electricity from its accounting and environmental attributes, the transition to renewable electrical energy would be generally applicable for almost all organisations. This enables hospitals to support and claim renewable electricity through market-based instruments, irrespective of the physical electricity mix delivered through the grid. Although radiology departments are often embedded within larger organisations, such as hospitals, that are responsible for energy procurement, they can still influence procurement decisions by supporting the transition to renewable energy sources or by covering the additional costs associated with market-based renewable electricity for their share of energy consumption. On-site renewable generation, such as photovoltaic panels, can complement these efforts.

As the GHG emission and primary energy conversion factors are broadly comparable across the main renewable electricity sources, especially when compared with fossil-based non-renewable electricity mixes, these findings are expected to be generally applicable to transitions from fossil-based non-renewable to renewable electricity mixes.

Carver et al demonstrated that MRI and CT services generate approximately 221 t and 108 t of CO₂e per year, respectively. The authors showed that energy consumption accounted for the largest share of emissions (58% for MRI, 33% for CT), followed by disposable supplies (26% for MRI, 16% for CT), capital equipment production (7% for MRI, 13% for CT), and linens (4% for MRI, 11% for CT) [16]. Therefore, they hypothesised that switching to solar photovoltaic electricity could reduce total emissions from MRI machines by 70% and from CT machines by 40%, with an increase in the relative impacts of scanner production, disposable supplies, and linens [17].

Common strategies to reduce the environmental impact of medical imaging include minimising direct energy use, such as turning off machines when not in use, shortening imaging protocols, and optimising scheduling to reduce scanner idle time [7, 18–20]. These actions are typically well-accepted as they also lower costs. However, our results show that switching to renewable electricity is the most effective strategy for reducing GHG emissions, particularly for major electricity consumers such as MRI and CT systems.

Our results also suggest that increasing the number of MRI examinations performed annually can significantly reduce primary energy consumption per examination by decreasing non-productive energy use outside operational hours.

Remarkably, after transitioning to renewable energy, materials and pharmaceuticals contribute the majority of GHG emissions. LCA studies confirm that materials, pharmaceuticals, and disposable supplies (e.g. medical linens and contrast media) significantly impact carbon footprints [5, 17, 21]. For example, previous studies have demonstrated that disposables account for up to 24% of emissions in radiography, 4–11% in MRI/CT [17], and 41% in the IR suite [22]. To address this, a dedicated model was developed to calculate sustainability data, as no comparable tools existed.

This study highlighted the lack of LCA-based sustainability factors, particularly for pharmaceutical products, although data is available for other sectors in established databases [13]. It is of utmost importance that the materials and pharmaceutical industries improve transparency regarding the environmental impacts of their products across the entire supply chain, thereby enabling more robust and reproducible sustainability assessments and supporting informed procurement decisions.

This study has some limitations. First, it was conducted as a single-centre study in the radiology department of a medium-sized Swiss hospital (approximately 200 beds), which may limit the generalizability of the findings to hospitals with different infrastructures, operational practices, or energy systems. However, the results could serve as a starting point for mitigation strategies in other hospitals, especially in industrialised countries. Second, some data were based on design specifications due to the impracticality or excessive effort required for direct measurements. However, these data were cross-checked with vendor manuals and publicly available sources to ensure accuracy and reliability, and this approach was applied only to elements with minor impacts on the overall results, so the conclusions remain valid. Another limitation is the exclusion of mammography machines, which are located in a separate hospital area. While their inclusion might have affected energy consumption and emissions, their contribution is expected to be minimal compared to major energy consumers such as CT and MRI systems. As a further limitation, measured electricity consumption data for the years 2023 and 2025 were available only for part of the year. Therefore, these values were annualised by proportionally extrapolating the recorded electricity consumption to a full year. This approach was considered appropriate because, in our case, electricity consumption, including cooling production, is only slightly influenced by outdoor temperature, as also demonstrated by the monthly data for 2024. Lastly, the use of AI to estimate non-public LCA values for materials and pharmaceuticals has inherent limitations. However, this was the only feasible approach, as LCA data are not publicly available for most products, and the environmentally extended input–output (EEIO) method was not suitable for our purposes (e.g. because it cannot distinguish between individual products). The AI agents were explicitly instructed not to rely on their training data but instead to base their estimates exclusively on citable sources referenced in the LCA reports. This substantially reduced the likelihood of hallucinations; any remaining errors were generally limited to misunderstandings, which were further minimised through manual verification. Furthermore, we compared results across similar products and between different AI agents to identify and resolve potential errors and other major inaccuracies. Collectively, these measures enabled us to exclude—or at least substantially reduce—the occurrence of hallucinations and misunderstandings. Based on the results of multiple calculation runs using different AI agents, we estimate a reproducibility of about ±30% for each type of material or pharmaceutical product, meaning that variability at the level of individual products should average out across aggregated groups. However, product-specific factors—such as transport routes, transportation modes, and the type of energy used during production—may still introduce variability in the results. Therefore, although our approach may be considered exploratory, the main findings remain robust and do not change substantially even under significant variations in the estimated impacts of materials and pharmaceuticals.

In conclusion, our findings support the transition from fossil-based non-renewable to renewable electricity sources in radiology departments as a key strategy for reducing GHG emissions. As healthcare systems increasingly integrate sustainability into clinical practice, decarbonising electricity supply should be considered a priority, complemented by the procurement of materials and pharmaceuticals with lower environmental impacts. This will require standardised, supplier-provided LCA data across the healthcare supply chain to enable meaningful comparisons and evidence-based purchasing. Future research should address current data gaps and develop practical standardised frameworks for incorporating environmental metrics into radiology practice and healthcare procurement.

## Supplementary information

ELECTRONIC SUPPLEMENTARY MATERIAL

## Data Availability

Data generated or analysed during the study are available from the authors, upon reasonable request.

## Abbreviations

AI: Artificial intelligence
CO₂e: CO_2_-equivalent
GHG: Greenhouse gas
IR: Interventional radiology
ISO: International Organization for Standardization
LCA: Life cycle assessment
WAN: Wide area network
