# Supplementary material for: Environmental impact of a radiology department: greenhouse gas emissions and primary energy accounting and strategies for sustainable practice
Source: Insights Imaging. 2026 Oct 15;17:260. doi: 10.1186/s13244-026-02436-9 (PMC13672723; doi:10.1186/s13244-026-02436-9)
Supplement: Supplementary file 1 [file 13244_2026_2436_MOESM1_ESM.pdf]

Environmental impact of a radiology department: greenhouse gas emissions and primary energy accounting and strategies for sustainable practice.

ELECTRONIC SUPPLEMENTARY MATERIAL

Supplementary Figure 1. An example of a typical prompt used to request Google Gemini a detailed calculation / report (procedure performed for more than 70 products from the Materials and Pharmaceuticals category).

> *Radiology Department with MRI, CT, US, X-ray.*
>
> *Calculation of primary energy consumption and GHG emissions (Scope 1-3) for ProHance 10 ml sir, ProHance 17 ml sir, ProHance flac 10 ml, ProHance flac 20 ml.*
>
> *Package with polymer, not glass.*
>
> *With API production. With Feedstock energy of the basic materials which go to the incineration, including the energy and emissions of the extraction of the basic materials. Including oxidation phase during usage for contrast agents and other liquid products. Cradle-to-grave. Transport to Bellinzona.*
>
> *Robust audit level.*
>
> *Output = Report*
>
> *Add Prompt, AI model/version/thinking model (Gemini...), day and time. Answer in English.*
>
> *Plan your proceeding and rethink.*
>
> *Create an in-depth analysis of at least 1000 words and explicitly cite at least 10 different primary sources.*
>
> *Consider statements without explicit source references to be incomplete. Every factual claim must be substantiated with an inline link or a footnote.*
>
> *At the end of each section, create a list of sources used in the following format: [Title](URL) - Date accessed.*
>
> *Do not use general information from your training data set. Rely exclusively on the current web sources found during this deep research.*
>
> *At the end of the report there has to be a table with for every element on the attached list the Total of primary energy and the Total of GHG emissions.*

Supplementary Figure 2. Extract from one report created by Google Gemini containing the LCA calculations in accordance with ISO 14040

Life Cycle Assessment and Environmental Impact Report: Magnevist 2 mmol/L in Polymer Pre-filled Syringe

Executive Summary

This comprehensive research report provides a robust, "cradle-to-grave" Life Cycle Assessment (LCA) of the radiopharmaceutical product **Magnevist (gadopentetate dimeglumine) 2 mmol/L solution**, specifically packaged in a **20 mL Cyclic Olefin Polymer (COP) pre-filled syringe (PFS)**. The assessment is explicitly tailored to the operational context of a **Radiology Department in** [redacted] incorporating local logistical parameters and the specific waste management infrastructure provided by the [redacted]

The analysis adheres to the rigorous standards of ISO 14040/14044 and the GHG Protocol Product Standard, evaluating Primary Energy Consumption (PEC) and Greenhouse Gas (GHG) emissions across Scope 1, 2, and 3. The study addresses the user's specific requirement to evaluate a **polymer-based primary packaging** configuration as an alternative to traditional Type I borosilicate glass, quantifying the environmental trade-offs associated with this material substitution.

Key Performance Indicators

The calculated environmental burden for a single functional unit—defined as one 20 mL syringe delivered to Bellinzona, administered, and incinerated—is summarized below. These figures represent a synthesis of feedstock energy, manufacturing intensity, transport logistics, and end-of-life valorization.

**Impact Category**	**Value per Unit (20 mL Syringe)**	**Primary Driver**
**Global Warming Potential (GWP100)**	**0.148 kg CO_2e**	Polymer Resin Production & Incineration
**Primary Energy Consumption (PEC)**	**2.95 MJ**	Feedstock Energy (Oil/Gas for Polymer)

Supplementary Figure 3: Model of the tool for calculation of CO2e emissions and primary energy consumption of the radiology department, including major imaging equipments, building infrastructure, information technology, materials and pharmaceuticals.

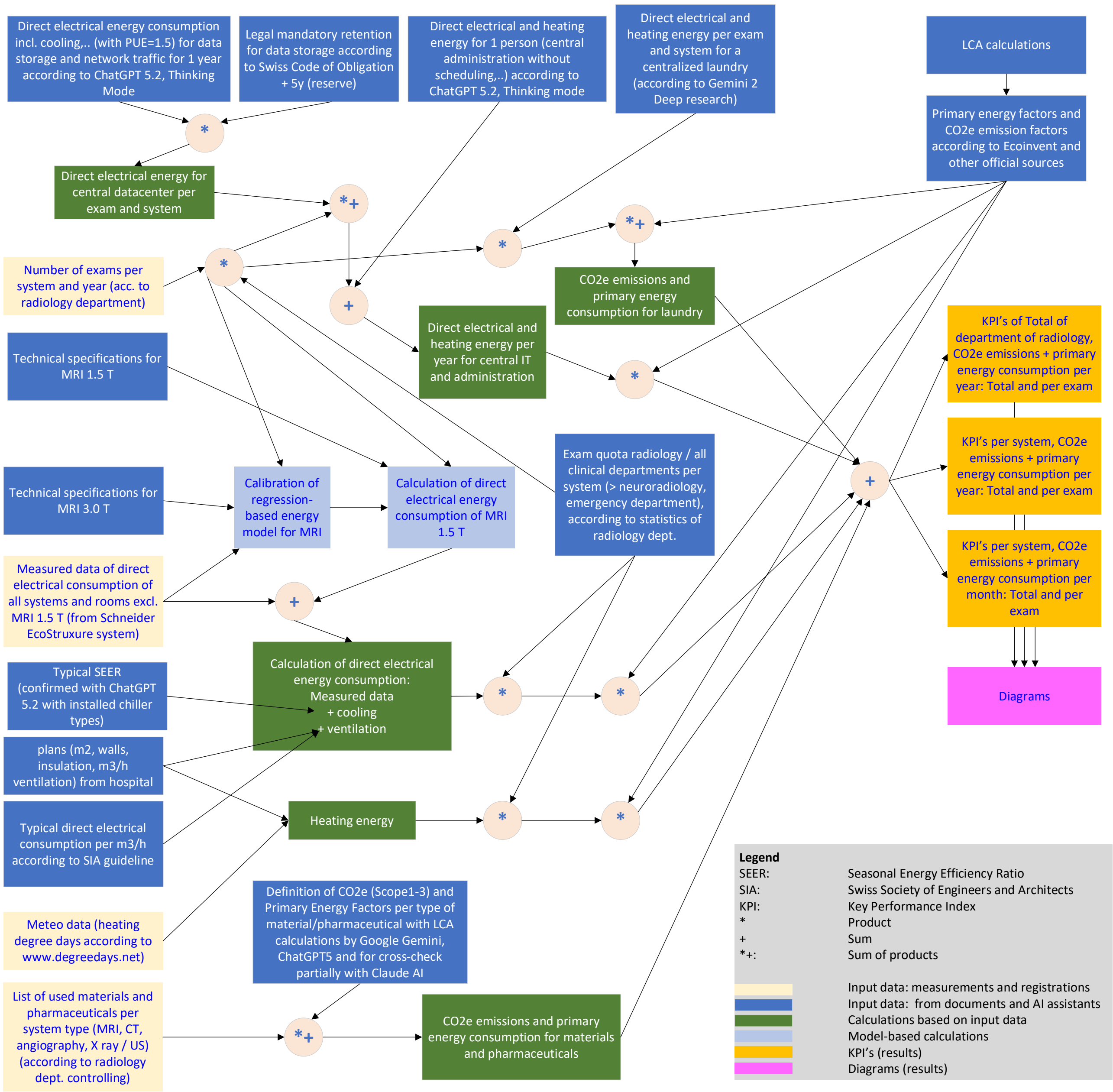

Supplementary Figure 4 The bar chart shows the primary energy consumption of the radiological department. Following the change from coal-based electricity products (year 2023) to renewable energy (years 2024 and 2025), the main electricity consumers (MRI and CT systems) showed an important reduction in primary energy consumption.

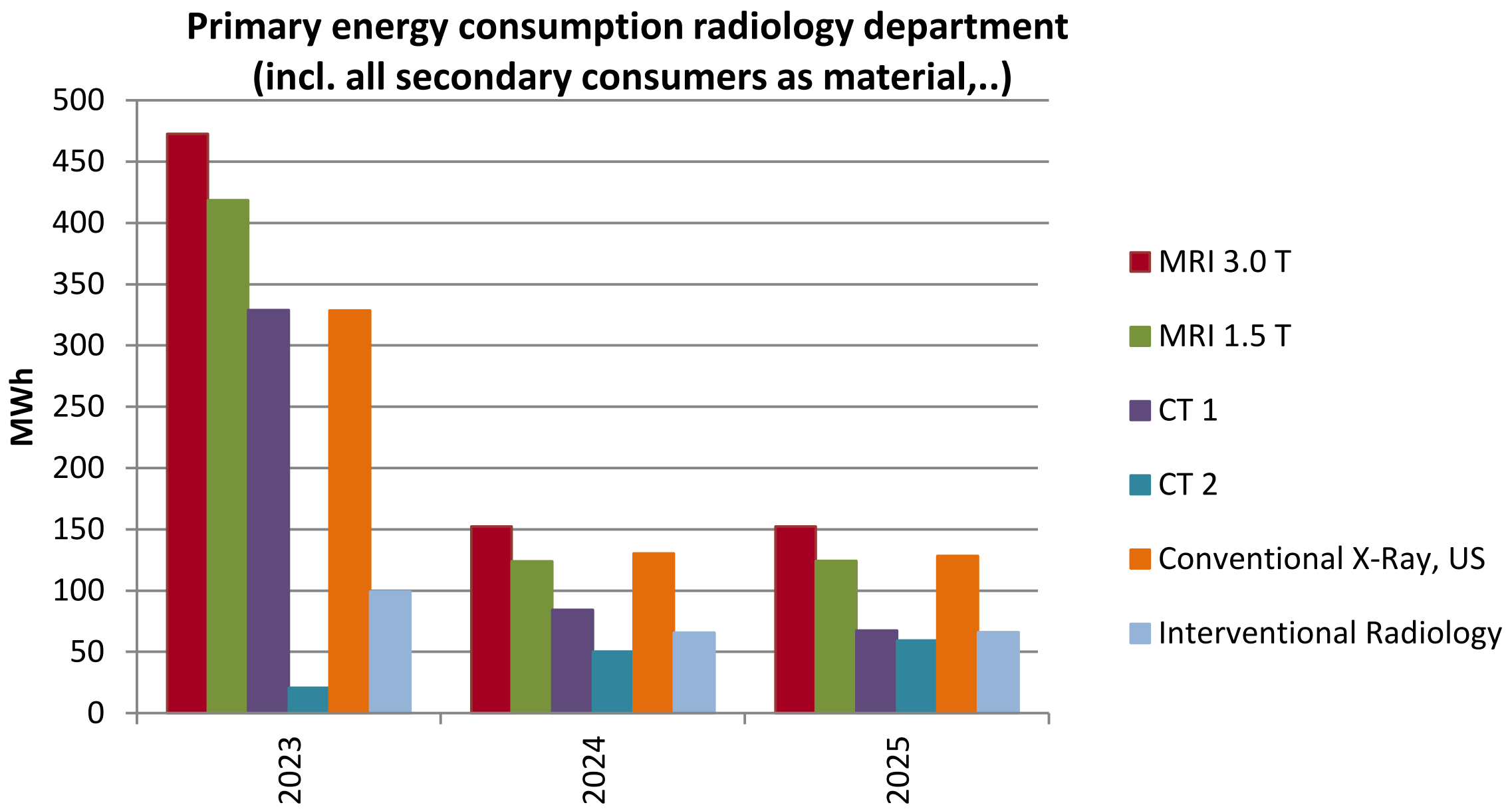

Note: CT2 was installed in 2023.

Supplementary Figure 5: The diagram shows the daily average power of weekdays, separated in the operational time and the nighttime of the MRI 3.0 T.

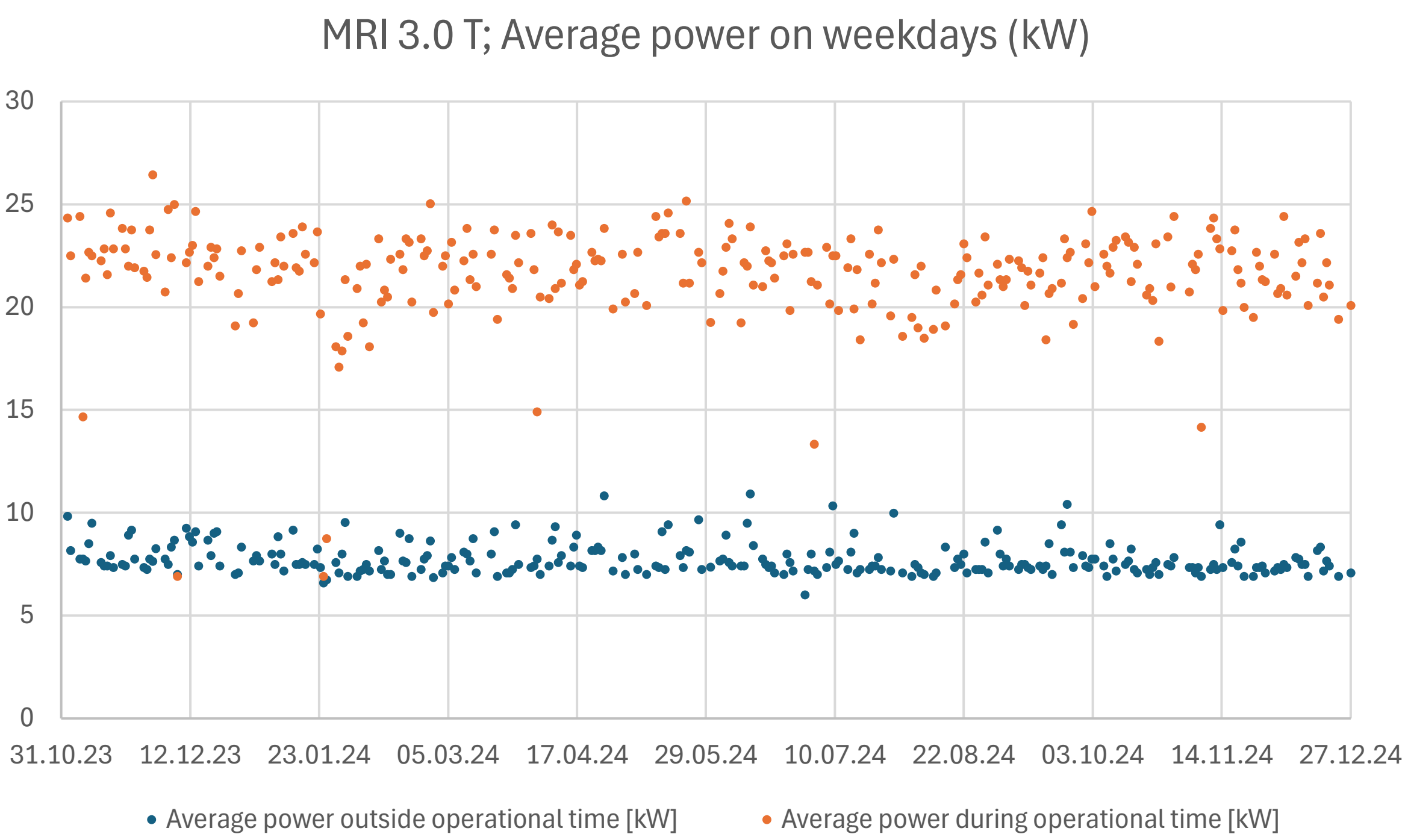

Supplementary Figure 6: The diagram shows that the direct electrical energy consumption of the MRI 3.0 T showed no substantial variation across the months.

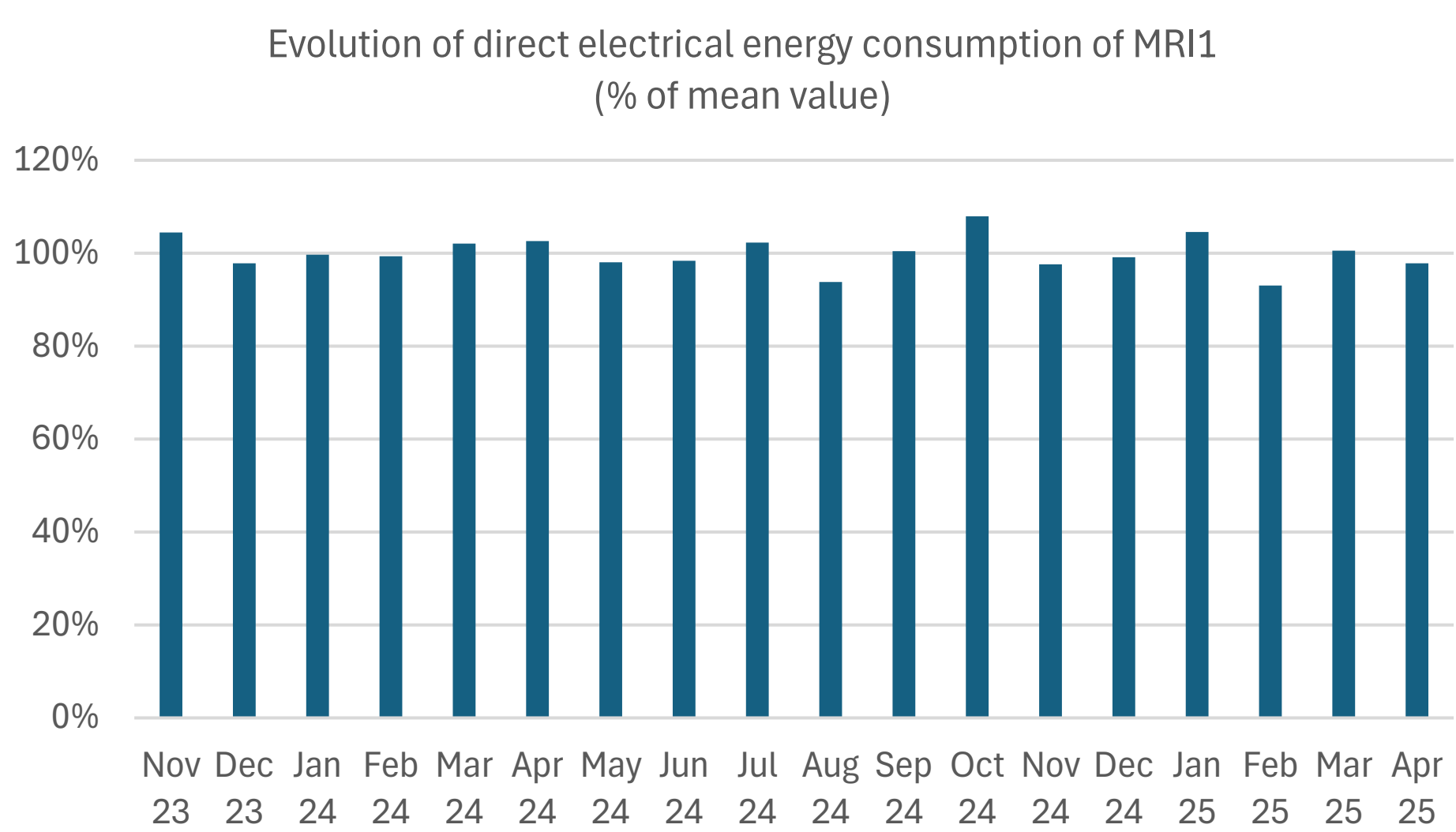

Supplementary Table 1: The table shows the primary energy and GHG emission factors of the key materials and pharmaceuticals, together with their contribution to the total material- and pharmaceutical-related primary energy consumption and GHG emissions. The items representing more than 1.3% of the total GHG emissions are shown. The same calculations were performed for all remaining materials and pharmaceuticals, comprising approximately 800 additional items. These calculations include the entire life cycle of each material and pharmaceutical, including raw material extraction, production, transportation, use, and end-of-life processes such as incineration and disposal.

Key Materials and Pharmaceuticals Driving Energy Consumption and GHG Emissions	% of Total Material- and Pharmaceutical-Related ...			Unit	Cradle-to-Grave, incl. Feedstock	
	Primary Energy Consumption	GHG emissions			Primary energy factor (MJ/unit)	GHG emissions (kg CO2e/unit)
CD Primeon Watershield x Discpr PP-100	1.91%	1.78%		CD	5.25	0.33
Non-sterile nitrile gloves, size L	2.42%	1.54%		Box	95.20	4.03
Universal radiology set 06/29343	7.27%	8.34%	Specifically prepared for our hospital.	Set	66.80	5.10
Disposable protective drape for table, 210 × 80 cm	2.84%	4.51%		Drape	2.74	0.29
Disposable cover for MRI headphones	1.54%	1.66%		Cover	0.60	0.04
Accupaque 300 vial 100 ml	1.38%	1.78%		Vial	24.50	2.10
Angiography set 411004/03	1.85%	1.97%	Specifically prepared for our hospital.	Set	170.00	12.00
Angiography procedure pack (Body PC Pack)	4.14%	4.39%	Specifically prepared for our hospital.	Set	170.00	12.00
Accupaque 350 vial 200 ml	2.07%	2.69%		Vial	48.50	4.20
Accupaque 350 vial 500 ml	22.30%	28.91%		Vial	105.50	9.10
CT Express patient-to-injector connection set 1056503	10.88%	6.74%	Specifically prepared for our hospital.	Set	10.80	0.45
**TOTAL**	**58.59%**	**64.31%**				
